# Electronic health records: Is it a risk worth taking in healthcare delivery?

**DOI:** 10.3205/hta000123

**Published:** 2015-12-10

**Authors:** Vera Lúcia Raposo

**Affiliations:** 1 Faculty of Law, Macao University, Macao, China; 2 Faculty of Law, Coimbra University, Coimbra, Portugal

**Keywords:** electronic health records, medical fault, technology, medical liability, patient safety

## Abstract

The electronic health record represents a major change in healthcare delivery, either for health professionals and health institutions, either for patients.

In this essay we will mainly focus on its consequences regarding patient safety and medical liability. In this particular domain the electronic health record has dual effects: on one side prevents medical errors and, in this sense, promotes patient safety and protects the doctor from lawsuits; but, on the other side, when not used properly, it may also generate other kind of errors, potentially threatening patient safety and, therefore, increasing the risk of juridical liability for the physician.

This paper intends to underline the main human errors, technologic mistakes and medical faults that may occur while using the electronic health record and the ways to overcome them, also explaining how the electronic health record may be used in court during a judicial proceeding.

## 1 Introduction

The Electronic Health Record (EHR, including patient electronic medical files, electronic prescriptions and electronic guidelines for medical support) is a modality of medical record that is not confined to store medical information about the patient, as the traditional medical record. Conversely, the EHR allows an all new range of possibilities, such as to analyse and to compare the various results of exams and other data, resulting in a truly mechanism of information management, aimed to promote efficiency and speedy solutions. It also includes reminders, alarms and guidelines, transforming the content of healthcare decisions. In addition, the EHR makes possible computerized prescriptions and computerized healthcare instructions, as well as improves the communication within the medical team. The improvement in communication with distant healthcare professionals, and even with patients, opens the door to telemonitoring and to other forms of telemedicine.

For the above-mentioned reasons, the implementation of the EHR in the majority of hospitals and clinics during the last decades caused a massive modification in the way healthcare is delivered. It would be very simplistic to say that it merely involves replacing sheets of paper by its electronic form. In fact, when analysed altogether with some other technological modifications that are connected with it, such as the communication between doctor and patient by e-mail, telemedicine, and medical apps, it translates in a truly Copernican revolution in healthcare. In consequence, it changes the relationship between elements of the medical team, as well as the classic doctor-patient relationship.

In the present paper we will analyse the following issues:

1. the consequences (positives and negatives) carried by the introduction of EHRs in health care delivery;
2. how their use can influence medical fault (namely, if EHRs can increase or decrease medical faults);
3. the way EHRs can be operated in order to promote patient’s safety;
4. the standard of care requested from health care professionals dealing with EHRs.

The final aim is to evaluate how the introduction of EHRs is changing health care delivery.

## 2 Electronic health records: the good and the bad

Despite the undeniable benefit of the EHR, some concerns must be held in consideration in its evaluation, especially because, at the present moment, we still lack regulatory requirements to evaluate the EHR’s efficiency and safety, nor there is an efficient mechanism to hold manufacturers and users accountable for the injuries that may be involved in its use.

### 2.1 The good

The advantages of the EHR have been pointed out by many scholars and entities [1], [2].

The speed in accessing the information and the amount of data accessible, especially in a very short period of time, are some of the main features of the EHR, allowing the medical team to have access to information that otherwise would go unnoticed, with the additional benefits of easily filtering that information according with the chosen criteria of research (by episodes, by date, by drugs). The simplicity and efficiency that result therefrom allow rapid methods to relate the recorded data in order to, e.g., identify drug’s incompatibilities or situations that may cause the patient’s relapse. Differently, when using the traditional medical record, it is frequent to have a great pile of paper stored in different facilities and services, easy to get lost or destroyed, thus, leading to medical errors based on the lack of information in due time.

To this extent, the EHR helps prevent medical errors. For instance, it allows a more accurate calculation regarding the dose of the prescribed drug; it makes possible to predict the consequences of interaction with other medicines; and it guarantees more accuracy in data calculation, such as body mass index for anaesthetic effects. 

Some systems even contain programs that warn about potential risks to the patient (stroke, drug allergies), which serve as reminders for cases in which the doctor could miss out an important clue. As pointed out in a paper from Harvard Medical School [3], ‘there is broad consensus that electronic health records are an essential foundation for the delivery of high quality care. As electronic health record adoption proceeds as a national health policy objective, some have wondered whether EHRs can help to prevent medical malpractice claims’.

It also presents benefits regarding the information that should be communicated to the patient, since it offers a substantial list of written information on his medical condition, effectively adapted to his situation, together with the necessary precautions to be taken, such as diet and drug dosage, which can simply be printed and delivered to the patient.

Furthermore, the provided information is easier to read and to understand, without the problems of calligraphy, regional specificities or some doctor’s particular expressions, since language is standardized. 

In addition, the EHR promotes and facilitates teamwork. On the one hand, because it allows more than one person to work simultaneously in the clinical file; on the other hand, because it allows interconnectivity with other agents, not only other members of the medical team, but also with laboratories, pharmaceuticals, and other hospitals, and even establishes the basis for telemedicine and patient monitoring from a distance. It is so because the EHR ensures the mobility of data from one service to another, or – if allowed by the personal data protection schemes – from one institution to another. Doctors may even access data by remote control, therefore, allowing the patient’s follow-up from home, or from another part of the world.

The communication of information between several health agents also prevents the patient submission to repeated examinations, sometimes painful and dangerous, particularly in what concerns the unnecessary repetition of tests that the patient probably had recently been subjected to. Another advantage regards the fight against the waste of medical resources, an especially important target if we have in mind that medical exams are usually costly and, *et pour cause*, not immediately available to all that need them.

While computerization raises many problems in terms of privacy, on the other hand it solves some issues regarding private data protection. Note that when the medical record on paper is used for administrative purposes (e.g., billing and accounting), usually the administrative staff has access to all its content, because the information that they ultimately need is mixed with other data that should be private, such as health and genetic data. By contrast, in its computerized form it is possible to create different profiles and different levels of access, so that the staff only has access to the specific information they need, and not to sensitive patient’s data.

It may carry so many benefits that authors such as Hoffman and Podgurski [4] proposed a project of financial support from governments in order to achieve the general adoption of EHR, which may be a good incentive to adopt it, especially because of the financial burdens involved in its implementation.

### 2.2 The bad

Despite the many advantages, let’s not forget also the EHR’s risks – known as e-iatrogenesis [5] – many of them the opposite of the referred potentialities [2], [6], [7], [8], [9], [10], [11], [12].

Although technology in its current state is very reliable, it is still not without dangers, from computer bugs to cyber-attacks that can leave the system inoperative or cause functional errors, some with serious consequences.

The mere loss of a password is enough to involve problems in system operation, since it prevents the use of the EHR and its information, eventually precluding the provision of adequate medical care. 

Even when the system is operating ‘normally’ (i.e., with no virus or cyber attacks), it may lead to some errors caused by the software design itself. E.g., we have the case of a patient, whose treatment for cancer was delayed for several years, because, instead of referring the doctor to the last exam performed, the system referred to an older normal exam which did not present any abnormal results.

Hoffman and Podgurski [2] relate different episodes of medical injuries caused by computer errors, such as when the EHR software of the Veterans Affairs led to the administration of potentially dangerous medicine’s doses. Actually, the majority of patient’s injuries caused during the use of EHRs come from wrongful ordering and administering of drugs or peroneus diagnosis originated by the lack of the necessary information by the EHR to make the proper diagnosis.

Those mistakes are, in a way, to be expected. In fact, the EHR has become so complete and complex that the technology underneath is, likewise, quite complex. A small flaw can throw it all away, by messing with the records of many patients (adding, dealing, or misplacing data).

Another difficulty to be considered relates with the possible simultaneous existence of two medical records for the same patient, a computerized one and another in paper format, a frequent situation in the beginning of the EHR implementation, so that a patient will have a record on paper referring to past events, and another one in electronic form for future events. However, this duality weakens many of the advantages aimed by the EHR in terms of efficiency and error prevention and may even cause confusions and malfunctions. In *Johnson v. Hillcrest Health Center, Inc.* – 70 P.3d 811 (Okla. 2003) [13] – the doctor sent the patient home twice alleging that his condition was not serious, but the patient ended up dying in another hospital of heart attack because his heart condition was not diagnosed in due time. In court, the claimant, Mrs. Johnson, wife of the deceased, alleged that the doctors and the hospital failed in storing the results of the exams, which were placed in the wrong chart, so that the doctor did not find them. However, the doctor could have traced them in the system, what he did not, since probably he was used to solely verify the paper chart. Though the doctor settled the case, the hospital did not, so, the court had the chance to analyse the behaviour of health institutions that allowed the parallel existence of a medical file in paper, and another one computerized. The court stated that, in this case, the applicable standard of care demanded the hospital to include all patient information not only in the computer, but also in the paper chart. 

On the other hand, technology may exacerbate the error. In effect, it is a well-known fact that many users of the EHR simply make copy/paste of past records of the patient, and even of records of other patients, in order to satisfy the demanding information request made by the system. But the simple fact of copying information from one record to another multiplies mistakes, because an eventual error in one record turns into dozens of errors in dozens of records. 

In addition, the copy/paste procedure promoted by the EHR may raise some questions in a litigation context, because the repetition of exactly the same information in the clinical records of different patients, or in different appointments regarding the same patient, will be a good indicator of illegitimate record. 

The demand for too much information, and the actual possibility of inserting too much of it, leads to another problem: the excess of data, most of which irrelevant to access the patient clinical state, and that can actually jeopardize the doctor’s task in evaluating pertinent information, since he might simply get lost in so much data. 

Privacy breaches are another relevant concern. Health and genetic data are very attractive for many industries, so, hackers may intrude in the system in order to get those data. This is the reason why laws all around the world are very restrictive in what concerns those data, and impose several penalties; not only to the ones that unlawfully access the data, but also to the ones in charge of protecting them; in our case, the doctor and/or the healthcare institution.

Another negative aspect is the cost involved in the implementation of the EHR, not only with what regards to the software purchase, but also its installation, maintenance and proper training of its users. These costs continue even after the initial phase, since software update, breakdown arrangements and knowledge apprises will systematically be requested.

Finally, the risk of a medicine more concentrated in the computer than in the patient is a very real one. Doctor-patient relationship may become impersonal, since the doctor will spend most of the consultation typing on a keyboard, without even looking at the patient, a behaviour that, in turn, will seriously affect doctor-patient relationship, especially in terms of informed consent.

The risks involved may become so stringent that they led some authors to claim that litigation might rise for doctors using EHRs and, as a result, insurance companies will increase the awards, specifically for higher risk specialities. This is the case of Ozeran and Anderson [11], relying on the study ‘Medical Professional Liability in a Changing Healthcare Environment: The New Story Unfolds’, presented by the Conning Research and Consulting in 2010. However, other studies defend otherwise. For instance, Mangalmurti, Murtagh and Mello [10] argue that the trend is for insurers to lower their premiums for policyholders who use EHR, precisely because it is considered more reliable than the paper one. Therefore, we still don’t have enough studies and data in order to make a credible forecast about the consequences of EHRs in the future of insurance premiums and litigation. Eventually, the consequence will be dictated by the outcome of lawsuits involving EHRs, all depending if courts will find them a good support for healthcare delivery or, on the opposite, a dangerous instrument regarding the standard of care, which, in turn, will depend on the way how institutions and healthcare professionals deal with the EHR. In our opinion, and as it will be further developed, EHR can actually become a very useful instrument to promote patient safety and to avoid medical faults, but, in order to do so, its proper use is absolutely required.

## 3 EHR and medical faults

### 3.1 Medical faults promoted by the EHR

Some studies refer that litigation increased through the use of the EHR, almost leading to believe that this mechanism promotes medical faults. But, in fact, some of those studies refer to the first years after the implementation of the EHR – see the study of Weir [14] about the experience of the Veterans Health Administration's computerized patient record system –, when the lack of experience in its handling and its technological degree of development was still in its very beginning, and it is a well known fact that novelties usually foster litigation. The fact is that nowadays almost every study [15] underlines the fact that the danger does not lay in the EHR itself, but on the misuse made by unprepared users.

Actually, we believe that litigation surrounding the medical activity increased because of the new perceptions on the role of healthcare professionals and medicine, firming the (erroneous) perception in society that scientific and technological developments can solve and heal everything and, thus, turning much stricter the standard of care. Therefore, it is unfair to attack the EHR for increasing the number of medical liability cases, a circumstance due to multiple factors, under which the EHR plays a minor role. 

As stated above, the EHR is not, *de per se*, a new source of medical faults and lawsuits, placing doctors in an higher risk of getting sued. Quite the opposite, it is likely that the generalization of the EHR will turn its use in the best medical practice for healthcare professionals, in such a way that it would be precisely the maintenance of the old paper medical file that would force the doctor to justify why he has not already adopted the EHR. In other words, in the near future, the adoption of the EHR will probably be the standard of care expected from health institutions and healthcare professionals, therefore, helthcare providers risk a conviction whenever a patient suffers any injury while being treated using the paper file as resource [10], [13].

But it is a fact that the EHR has some risks, which may generate new kinds of medical faults. Curiously enough, some of those risks result precisely from its benefits. 

In effect, EHRs allow the access to extensive information in a few seconds, and the fact is that too much information can overload the physician and lead him to lose the file’s essential content. To overcome this difficulty Hoffman and Podgurski [2] suggest that the doctor could require a nurse to summarize the most relevant notes from the wide range of information about the patient. However, this solution would allow the nurse to access information that probably he or she is not authorized to, since privacy laws in this matter tend to be very restrictive. In addition, according with the rules on tort liability, chances are that doctors would be held liable for any mistake occurring during the nurse’s summary, even if they didn’t have any participation on it whatsoever, therefore, one may assume that doctors would rather analyse the records themselves.

The already referred alarms aimed to identify patient’s risk (e.g., from drug interaction) may also turn from a benefit to a peril when they become too frequent – because they operate motivated by a very low level of risk –, therefore, too banal. Their excessive repetition will lead the physician to disregard serious risks for the patient, risks that perhaps he would have noticed by the traditional methods of human evaluation of information [16]. 

The coded language used by the system, which was just qualified as a benefit, also raises problems. First, it is necessary to know and understand it. Furthermore, that language may prove too standardized to describe accurately the clinical status of a particular patient, with all its intricacies and peculiarities, which may be decisive in the outcome of the case. Another difficulty to have in mind relates with the fact that the choice of any code presupposes a previous diagnosis, which often cannot even be done. In addition, it can be difficult to choose the correct code, especially in more complex cases or when some of the codes are similar in what regards the situation they describe. Likewise, we also cannot set aside the hypothesis of human error in choosing the code, which will often be the case, as data appears many times as mere strings of numbers or letters aligned together in a small computer screen.

The very way in which the record is done – *rectius*, can be done – promotes medical error. The reason is that the system asks for very detailed information, that often is not available to the clinician. The requirement to fill in exhaustively various fields, together with the parallel availability of similar information in a table in the same screen, makes techniques of copy/paste very seducing and hard to resist when the system asks for information and doctor has little time to provide it [17], [18]. The problem is that information that was valid for a prior date may not be adequate for any other time, and it is a well-known fact that the quality of healthcare largely depends on the integrity, reliability and accuracy of health information. The fact that the same data (the same answers, the same values) are carried over from week to week, fosters errors, since the medical team does not realize that the clinical condition of the patient has changed and instead, continues to reason based on outdated information.

Another peril comes from the templates offered by the EHR. Of course that they turn record much easier, since it is all about crosses and checks. But the fact is that many records can end up having the same content, disregarding the particularities of each patient, sometimes unable to fit a standardized template. Moreover, some systems automatically fill the empty spaces without the doctor noticing it, once again discounting the specificities of the patient [6]. Besides, the speed at which everything happens, when it takes simply a click to change completely the information on the computer screen, decreases attention in data visualization. 

Another threat may come from the doctor’s reliance on the outcome of recent tests carried out previously by him or by another doctor. It is a fact that the EHR promotes easy access to the result of those tests, whereas in another situation the physician would tend to duplicate them. Nonetheless, the clinical condition of the patient may have changed in the meantime, so, decisions based on previous results may become a present hazard.

Another dimension of the problems raised by easy access to previous exams occurs when the first examination is carried out by a certain doctor, who wrongly recorded the results, and another doctor came to make decisions and take actions based on those erroneous results. When this scenario occurs we can have a litigation snowball: the patient triggers the doctor who performed the medical act on him, who, in turn, turns against the doctor who did the initial misleading record. It is true that incidents of this nature may also occur with paper-based medical files, however, in this last scenario those incidents are rarer and with limited effects, precisely because one of the disadvantages of paper records is the difficulty in gaining access to previous patient information; while within the EHR any existing error propagates its harmful effects very quickly, given the easy access to information.

Some medical faults may be generated by the way EHR systems make information available to the doctor. Usually the screen presents a list of small letters and numbers displayed in a column, thus, the doctor may easily select the wrong patient name or the incorrect medication among the long list of small letters. It is also conceivable that the information will eventually be recorded on the wrong patient record, which, besides providing incorrect clinical decisions, or even death, can cause serious and unnecessary distress to a patient who was been reported of suffering from a disease that, after all, does not affect him.

Some errors can be generated by programs that the doctor, on its own initiative, and often without authorization, installs on the computer, and that may conflict with the normal operation of the software. Therefore, this type of conduct shall be forbidden to users, otherwise it may distort the whole mechanism. If through this adulteration the patient suffers any damage the liability will fall on the installer of the clandestine program, and perhaps on the hospital, and/or the manufacturer, for failing to take the necessary precautions to prevent it.

As already noted, sometimes are the (apparent) EHR advantages that become its greatest enemies. E.g., some software have already embedded a pack of guidelines that advise the doctor about what to do in a given situation. Surely this additional service greatly facilitates medical treatments. But sometimes it may be an inadequate instruction, since those guidelines are laid down for the majority of cases, reasoning in the abstract, without taking into account the patient particularities. However, the doctor may be tempted to follow what the system recommends, without considering whether, in the particular case, this is the most appropriate conduct. In fact, the spread of technology in healthcare is reaching decision-making itself and actually doctors are allowing machines to make some routine decisions, either because of lack of time, or because they believe the machine is a better decision-maker. For instance, it is common to have a prescription based solely in the registered symptoms and its informatic evaluation, which, of course, can originate wrongful decisions, because human factors and specific particularities are not considered. The temptation to follow the system’s advice, without further analysis, comes especially from less skilled or less experienced clinicians, the ones that are not so confident in themselves or that are overwhelmed with work.

Furthermore, the system frequently operates with reminders based on negligible levels of risk, which makes the alarm become so frequent that turns out to be disregarded by the physician, including in situations where the doctor would have detected a problem if this mechanism did not exist [19].

The main consequence of the concretization of these risks is that, when comparing with the risks involving paper medical files, here any negative effects are intensified and may potentially involve a large number of people. 

### 3.2 The use of the EHR in a court of law

One of the concerns to take into account when purchasing an EHR software consists in choosing an operating system that not only is effective in medical terms, but it is also useful in the event of litigation [11].

Once there is the possibility of the EHR to be used in court, potential or actual defendants may be tempted to modify the record contents to hide eventual medical faults [20]. However, the computerized form easily allows the detection of these modifications, the time when they were made and by whom. Admittedly, the paper file also allows this kind of manipulation, since it is easy to wipe documents, delete notes in the margins or, rather, add them, as some sad judicial episodes have shown. But these manipulations are relatively easy to identify. E.g., if the handwriting of a note is different from the rest of the document, or it is written with a different colour, those facts soon generate doubts as to its veracity or the timing of the respective insertion. At this point the EHR have some special features. On one hand, it is not always possible to make such additions or deletions, to the extent that some programs do not allow subsequent corrections. On the other hand, interferences of this type may require more technical knowledge than the mere addition of a note or the deleting of a document, actions that in the paper record can be undertaken by anyone with physical access to the file, while handling the EHR not only requires passwords, as some specific knowledge. But, above all, the point is that in the clinical process on paper many manipulations may go overlooked and even when identified it is almost impossible to ascertain the author. Conversely, in the EHR all that is added, modified or erased leaves its track and it is likely to become notorious through an audit (thus, it is foreseeable that the generalization of EHR will lead to the presence of informatics experts in court during medical malpractice disputes), which allows to identify the existence of any manoeuvre (or even manoeuvre attempts) and, more than that, to identify those who carried it out (except for the case where there is misuse of password, but in those scenarios the responsibility is assigned to the holder of the password, which can be held liable under his own negligence in guarding the same).

The audits may also be an important tool to confirm for how long did the doctor analyse a certain exam or any other document, by checking when did he log in and log off and how much time was the information displayed on the computer screen. For instance, if the doctor sustains that before performing a certain surgical intervention he was careful enough to analyse the results of an x-ray, but the system shows that the holder of the doctor’s username and password did not look into that file, his argumentation will certainly collapse very quickly. The same will happen if the doctor has in fact accessed the document, but only for five seconds, which will hardly sustain the defendant’s argument that he thoughtfully analysed the results of the examination [11].

These audits shall also verify whether the system alerted the physician to an occurrence and if the doctor ignored the warning, leading to conclude that the doctor moved away from the best standard of care [16]. Admittedly, warning systems that are too frequent become banal and may decrease or even annul the doctor’s negligence, thus, providing a defence for the charge. If it is set that the frequency of the alarm endangers patient safety, either the software manufacturer, or the buyer, may be held liable: the former because he produced a product that may become risky, since it induces in error; the latter for buying a product recognised as defective (inasmuch as it was previously declared as defective and dangerous).

Beside the reminders, the system often offers guidelines, which incorporate some *leges artis* in the software itself. The respect or disrespect for such guidelines will condition the defendant’s position in court. If the doctor acted in accordance with the guideline, this fact can be an evidence that he acted in a diligent way, except if the court considers that in the particular case the good standard of care would impose a different behaviour, based on the fact that the general rule provided by the system cannot embrace all the particularities of the specific situation. On the opposite hand, if the court confirms the guideline, the doctor would have to explain why he opted for a different conduct, as if a judicial presumption of negligence operated here, turning more difficult to the doctor to demonstrate his diligence.

In order to correctly assess all these elements it is crucial for the court to understand how the EHR works, its potentialities, and its fragilities. For instance, the knowledge of how a particular software alarm system works may be crucial for the court to assess the medical treatment, namely to know the frequency and level of risk adopted. So, the fact that an alarm has been ignored may indicate a careless behaviour, but if the court concludes that the alarm is too frequent, and likely to be ignored, the disregard of the alarm may become justified.

However, for many of these evidences to be examined in court, it is necessary that metadata (i.e., data about data) are considered admissible evidence, which is not possible in all jurisdictions [10], so the question remains to be clarified.

Another important fact about EHR files is that the court must have in mind that not all the information found in it at the trial would have been accessible to the doctor. In fact, the information provided by the EHR to the court can be misleading. Suppose that a damage is caused to the patient because the doctor was unaware of information that we know today, but that he couldn’t have known at the time, since it only came to be known in subsequent consultations. The system can provide all the information available at the present moment, but not the one available to the physician at the time the medical act was committed, while only the latter is relevant for assessing the legality and the diligence of his conduct.

## 4 Safety and efficiency

### 4.1 Cautions when choosing a software for EHR

The chosen software must meet different requirements. Its capacity to prevent medical error and the information provided in the event of litigation must be particularly accessed [20], [21].

First of all, it must be a duly accredited software, which undoubtedly conveys a certain degree of reliability. Accordingly, it is conceivable that the hospital will be liable if the damage suffered by the patient is due to the fact that the software does not perform adequately its functions [22]. Of course that the purchaser is not required to carry out a technical analysis of all products on the market in order to avoid disputes by wrong choice of the product, but if that software is known in the market for its poor quality certainly its choice reveals little diligence from the buyer [2]. The lawmaker should impose stringent regulations imposing standards to be met by software manufacturers, in similar terms to the existing regulation on drugs and medical devices. Actually, EHR software can probably be also considered a medical device, to the extent that likewise it is used in healthcare and, when defective, can cause serious harm to the patient, with the respective legal consequences. Actually, because of the similitudes between EHR and medical devices and drugs it can be argued that the notification system for adverse effects, existing for drugs and medical devices, could also be imposed to EHR, whether or not it is considered a medical device [2].

In what regards to the manufacture of EHRs, some authors suggest product uniformity, so that all EHRs would follow the same operating scheme and design, in order to facilitate the changing of software by healthcare operators, since the learning of a new operation mode can involve errors, hence, damages to the patient [2].

Patient privacy must be a constant concern. Therefore the system should be prepared to face possible cyber attacks. Another caution is that it should also operate through a hierarchical model of progressive disclosure of information through the use of profiles. The goal is to maintain control at all times over who accesses what and when. In order to perform this task the system must provide reports on the various accesses, dates and registration of any alteration of information or unauthorized access attempts. An unauthorized access situation can take place when one of the authorized users tries to access a level of information for which he lacks proper authorization (e.g., a nurse tries to access data only allowed to the doctor, or a third doctor tries to access data that only the treating physician has access), or when a stranger without any authorization tries to access the EHR. It is not always easy to identify the author of such hits, since sometimes stolen usernames and passwords are used. In the latter case what happens is that the improper user will enter the system using the identification of a certified user (theft of usernames and passwords). In other occasions there will be attempts to penetrate the system successively until the intruder “guesses” the right access-key, which requires the software to block after a number of failed ones.

Another caution to consider relates to the way the warning system operates. As mentioned before, some programs use alarms too often, because they function based on very low degrees of risk, thus causing alert fatigue. Take the concrete example of drug interactions: some programs run on long of lists of possible interactions, which causes virtually every prescription to trigger an alarm, leading the doctors to ignore them. So, it is recommended the choice of software that does not use extensive alarm lists. But chances are that such a software becomes increasingly difficult to find, since manufacturers tend to include quite exhaustive lists of ricks, precisely to avoid lawsuits grounded on the omission of alerts to an occurring damage [16], [23]. However, in the future presumably manufactures will be held liable on the opposite case, i.e., when the software works with excessive and disruptive alerts, turning them banal, thus, irrelevant and even counter-producing. The solution does not lie in the total elimination of alerts, given the benefits that result from them [24], but in reducing the frequency of warnings, in order to include only the more frequent hazards and the ones that, although rare, are especially severe, so to be effectively taken into account by the physician. Note that the suggested solution closely follows the one that has been advocated for the purposes of defining which risks the patient should be informed of regarding informed consent, which also points to the very common risks and to those rare but serious [25], [26], [27].

### 4.2 Cautions when using the EHR

The first step to prevent damage resulting from the use of EHRs is to be aware of the respective dangers and limitations in order to know how to manage them. It is required that users understand precisely how the system works, because many errors may be encouraged by the mere ignorance of the software’s functioning. Some of the models are more intuitive, others more complex, but all are operable, provided that the manufacturer makes training available and supplies appropriate information.

Secondly, it is imperative to issue guidelines (usually defined by the national medical association or by specific professional associations) regarding the correct conduct to adopt under certain circumstances, i.e., the appropriate and specific standard of care for healthcare professionals dealing with EHRs [2]. In fact, one of the most striking effects of the EHR is the imposition of a different, and more demanding, pattern of behaviour [13]. Actually, this is a normal consequence, expected each time that science and technology advance. The same effect occurred when science made it possible to offer diagnostic exams with a high level of predictability, and consequently law imposed on doctors an obligation of result when performing those exams, considering them negligent if the result obtained is not the correct one, because it is assumed that science developed to such a level that any erroneous result must be due to the doctor’s negligence, being up to the doctor to demonstrate that he acted diligently [25]. The same increase in the level of demanding is a consequence of the technological development represented by the EHR.

Besides these guidelines, each health institution should also formulate its own internal protocols, preferably grounded on the referred guidelines previously issued by professional associations. The aim is to establish the procedures to be taken in each different situation by their staff. Those protocols should define the proper use of EHR as how to record information about a patient and when to do so, how to consult the EHR correctly, how to prescribe medication or exams, how to prepare the informed consent and include it in the EHR, how to use the EHR to distribute tasks and communicate with other team members and how to manage EHR so that it can serve as a useful and reliable instrument of defence at trial. The internal protocols can also be very useful in clarifying how to behave in case of potentially risky situations, such as incorrect data recording, unauthorized access, data and identity theft operated through the system or unauthorized reports modification. Moreover, the protocols shall also define the conduct to adopt in case of unexpected incident, for instance the impossibility to access data about a patient that would be essential for certain medical act in an emergency situation, caused by a crack on the system or a virus, helpdesks that aren’t available and systems that are incompatible within the same service. In the event that some critical incident occurs, it must be investigated in order to identify its cause and create a guideline to deal with it in future occasions [28].

Either the guidelines or the protocols are essential in order to fully enjoy all the virtues of the EHR and, simultaneously, avoid its main dangers. But, in order to achieve those goals, guidelines definition cannot be the exclusive task of healthcare professionals. In fact, it is also necessary to have contributions from computer engineers and lawyers, since the correct management of the EHR demands the confluence of different knowledges.

## 5 A new standard of care

There are reasons to believe that in the near future the standard of care in medical practice will coincide with the use of EHR. Therefore, the practitioner or institution insisting in using the old paper file will be requested to explain in court why the EHR was not yet adopted, especially if the medical fault committed could be avoided by it. In parallel, it would be difficult for institutions not using EHRs to find doctors, precisely because of the increased burden on healthcare professionals not using EHR and the consequent risks of litigation.

This more demanding standard of care has many other features. For instance, the requirement of checking both the paper chart and the computerized one, just as can be deduced for the already commented Johnson case. Furthermore, courts will certainly be less tolerant towards missed information, since the EHR makes it possible to scrutinize everything with great celerity. Likewise, failures in communication amongst the medical team will not be tolerated in presence of a communication system that allows everyone to be connected and to share information at the same exact moment.

In sum, healthcare providers may be held liable for not using EHRs or for not take advantage of their potential.

## 6 Conclusion

Medical faults, human errors and injured patients always existed and, unfortunately, most probably always will exist. But the recognition of this fact does not prevent the search for new methods and mechanisms aimed to combat and avoid medical faults and patient injuries.

The EHR intends precisely to achieve this purpose, and in many ways it does prevent classical medical faults and errors. 

But, on the other hand, it would be naïf to expect the EHR to automatically decrease medical faults and improve patient safety. Actually, it may be quite the opposite, because it may become a tricky solution, especially when misused, since it may carry additional medical faults and be potentially threatening to patients.

Therefore, the solution does not lay in the coming back to the old paper clinical file, but, instead, in investing in EHR with better design and performance, better training for the EHR users and the definition of protocols and guidelines to orientate its correct use. 

In what specially concerns the effects of the EHR on medical malpractice litigation, it is plausible to assume that it will significantly increase the standard of care expected from healthcare providers and, in that particular sense, it can indeed become a meaningful risk.

## Notes

The article has been reviewed twice. The reviewers and the author do not agree on all points.

### Competing interests

The author declares that she has no competing interests.
